# Hepatocellular carcinoma complicated with huge posterior pancreas head lymph node metastasis and primary renal carcinoma: A case report

**DOI:** 10.3389/fonc.2022.989172

**Published:** 2022-12-08

**Authors:** Jun Chen, Zhiyi Zhou, Wenyan Chen, Abid Ali Khan, Zhikun Liu, Kai Wang, Fan Yang, Xiao Xu

**Affiliations:** ^1^ The Four School of Clinical Medicine, Zhejiang Chinese Medical University, Hangzhou, China; ^2^ Department of Hepatobiliary and Pancreatic Surgery, Affiliated Hangzhou First People’s Hospital, Zhejiang University School of Medicine, Hangzhou, China; ^3^ Key Laboratory of Integrated Oncology and Intelligent Medicine of Zhejiang Province, Affiliated Hangzhou First People's Hospital, Zhejiang University School of Medicine, Hangzhou, China

**Keywords:** hepatocellular carcinoma, primary renal carcinoma, lymph node metastasis, targeted therapy, immunotherapy, transcatheter arterial chemo-embolization

## Abstract

The incidence of hepatocellular carcinoma (HCC) associated with extrahepatic primary malignancy (EHPM) is extremely rare, especially for those that involve primary renal cell carcinoma (PRC). Here we present a case of a 66-year-old male who was diagnosed with HCC complicated with lymph node metastasis at posterior pancreas head and PRC. Biopsy results of the liver and the lymph node confirmed the diagnosis of HCC. The disease progressions of both HCC and PRC are controlled effectively following the initiation of comprehensive therapy including pembrolizumab, lenvatinib, radiotherapy, and transcatheter arterial chemo-embolization (TACE). Ultimately, the patient had successfully access to surgery and complete response (CR) of all the tumors were achieved after surgery.

## Introduction

Primary liver cancer is one of the commonest malignancies globally. Among the newly-emerged cases annually, about half occurred in China (1). Hepatocellular carcinoma (HCC) accounted for about 85-90% of primary liver cancer. It is reported that in China, it ranked as high as 4^th^ and 2^nd^ in morbidity and mortality rates among other malignancies and it is also the leading cause of cancer-related death in men aged 60 and below (2, 3). Despite hematogenous dissemination in HCC being relatively common, lymph node metastasis is uncommon when compared to other malignant tumors (such as lung cancer, gastric cancer, and renal cancer), which account for only 1.0% of patients who received operation (4). Furthermore, the presence of lymph node metastasis is considered a manifestation of advanced HCC and usually indicates a worse disease outcome. Surgical resection is the mainstay of treatment for early HCC and possesses a 5-year survival rate of 54.8% (5). However, early symptoms of HCC are often vague and non-specific until the disease progresses into the advanced stage. Also, the majority of HCC cases are usually complicated with hepatitis or cirrhosis, not to mention poorer functional hepatic reserve (FHR) at the late stage. Altogether, diagnosis at a late stage would render the patients non-eligible for radical resection. Therefore, a thorough clinical evaluation and individualized therapy are of utmost importance for HCC patients. We hereby report a case of a male patient who was diagnosed with HCC complicated with lymph node metastasis at posterior pancreas head and PRC.

## Case report

A 66-year-old male patient with a history of hepatitis B visited the community hospital in December 2021(baseline) due to yellow discoloration of the skin and the sclera, accompanied with right upper abdominal pain. The abdominal computed tomography (CT) scan revealed a mass in posterior pancreas head adjacent to IVC which suggested a malignant tumor, a hyperdense mass in right kidney, enlarged gallbladder, and cholelithiasis. Ultrasound-guided percutaneous transhepatic gallbladder drainage (PTGD) was performed upon admission but jaundice remained persistent post-operatively. The patient was then transferred to our hospital for further treatment.

**Figure 1:**
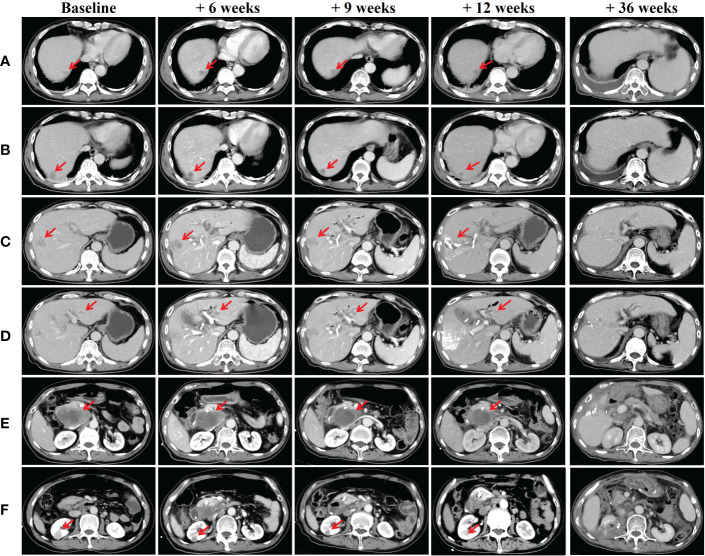
CT scans of the liver, posterior pancreas head and kidneys **(A-D)** Multiple intrahepatic space-occupying lesions; **(E)** Mass in posterior pancreas head; **(F)** Right renal mass. Abdominal CT scans were taken on baseline scan, baseline + 6 weeks (after first session: pembrolizumab and radiotherapy), baseline + 9 weeks (after second session: pembrolizumab plus lenvatinib), baseline + 12 weeks (after third session: pembrolizumab plus TACE) and baseline + 36 weeks (after synchronous partial resection of the liver and partial right nephrectomy).

On admission, physical examination revealed jaundice of the skin and the sclera accompanied with slight abdominal tenderness over the right hypochondriac region. No rebound tenderness or guarding was felt on palpation of the abdomen. Lab reports were as follow: AFP 9243.33 μg/L, PIVKA-II 897.6 mAU/mL, HBsAg (+), HBeAb (+), HBcAb IgG (+), HBV-DNA 8.81*10^2 IU/mL, Total Bilirubin 167.1 μmol/L, Direct Bilirubin 123.5 μmol/L. The enhanced abdominal CT scan was repeated which confirmed a mass measuring 8.0*4.5 cm at posterior pancreas head which was highly suggestive of malignant tumor. The scan also revealed multiple intrahepatic masses with dimensions ranging 1 to 3 cm, right renal mass which portrayed malignant feature, cholelithiasis, and cholecystitis (**Figure 1**).

**Figure 2:**
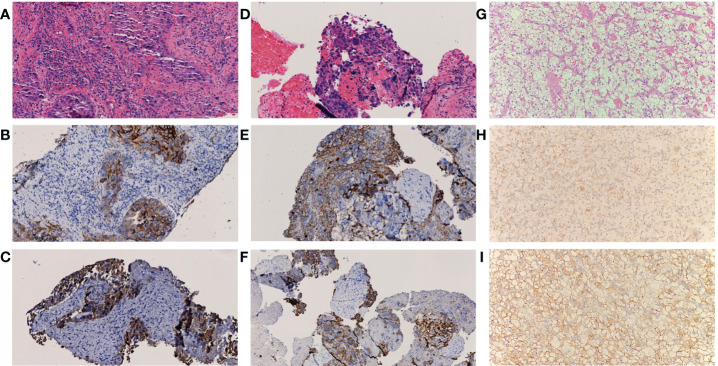
Pathological result of lesions **(A-C)** Histology of lesions in liver, **(D-F)** Histology of lesions in posterior pancreas head, **(G-I)** Histology of lesions in kidney. Both A and C showed atypical cell distributed in sheet manner (H&E staining, 200X); Immunohistochemistry result of samples in **(B)** GPC-3 (+), **(C)** CK19 (+), **(E)** Herpar1 (+) and **(F)** CK19 (+) indicate tumor cell differentiation of hepatic or hepatoid origin. Histopathological examination in **(G)** revealed clear-cell renal carcinoma, Furhman grade 2 with maximum diameter of 1.7 cm and negative margine (H&E staining, 200X); Immunohistochemical stain reveals positive marking result in AMACR **(H)** and CAIX **(I)**, indicate tumor cell differentiation of renal origin.

A multidisciplinary team (MDT) discussion was conducted and diagnosis was narrowed down to (1) posterior pancreas head neoplasm with multiple intrahepatic metastases or primary liver cancer associated with posterior pancreas head metastasis; (2) Primary renal cell carcinoma. To further confirm the diagnosis and alleviate the symptoms, endoscopic retrograde cholangiopancreatography (ERCP)-guided biliary stenting and biopsy were performed in the posterior pancreas head mass. BUS-guided liver mass biopsy was also performed and Pathology test result of the liver indicated a poorly differentiated HCC while the immunohistochemistry test result showed HepPar1 (+), Arg-1 partial (+), GPC-3 (+), AFP (+), CK7 partial (+), CK19 (+), ki-67 (+) 50% (**Figure 2**). Similarly, pathology test result of posterior pancreas head mass revealed a poorly differentiated carcinoma while the immunohistochemistry result showed HepPar1 (+), Arg-1 slight (+), GPC-3 (+), AFP Partial (+), CK19 (+), ki-67 (+) 50% (**Figure 2**). Pathology of the right renal mass was unavailable as renal biopsy was strongly denied by both the patient and his family.

Our team decided to adhere to the first-line treatment suggested by the National Comprehensive Cancer Network (NCCN) Guidelines for liver cancer since there was no definite target indicated for immunotherapy from the genetic testing. To minimize the effect of obstructive jaundice on PIVKA-II assessment, Vitamin K was supplemented immediately after the alleviation of biliary obstruction. Consequently, PIVKA-II level of the patient was successfully reduced to 64.01 mAU/mL after 10 days of vitamin K prescription which reflected that elevation of PIVKA-II was mainly due to biliary obstruction.

After the patient recovered from jaundice about 44 weeks ago (baseline + 2 weeks) (Total Bilirubin 43.3 μmol/L, Direct Bilirubin 33.2 μmol/L), combined therapy which consisted of pembrolizumab plus lenvatinib (pembrolizumab 200mg Q3W and lenvatinib 8mg QD) was initiated. Subsequently, radiotherapy for the metastatic lymph node posterior pancreas head was started 1 week after the combined therapy (Total radiation dose given was 30 Gy and fractionated into 6 doses of 5Gy each). 40 weeks ago (baseline + 6 weeks), repeated lab test results showed reduced level of AFP and PIVKA-II to 2173.04 μg/L and 63.66 mAU/mL respectively. Follow-up abdominal CT scan revealed multiple intrahepatic lesions which were dimensionally similar to the pre-treatment period. Nevertheless, the mass in the posterior pancreas head showed significant shrinkage and reduced enhancement intensity. The right renal mass was also smaller compared to the previous scan (**Figure 1**).

Pembrolizumab was administered for the second time about 40 weeks ago (baseline + 6 weeks) and post-therapy follow-up assessments were carried out about 36 weeks ago (baseline + 10 weeks). AFP and PIVKA-II levels were 1096.84 μg/L and 38 mAU/mL respectively. Overall shrinkage in all lesions was observed through CT scan (**Figure 1**). The third session of therapy which began 35 weeks ago (baseline + 11 weeks) included pembrolizumab and TACE. Post-therapy AFP and PIVKA-II levels were 249 μg/L and 76.28 mAU/mL respectively while other markers were within normal limits. Abdominal CT showed lipiodol deposition, and hepatic pneumatosis at segment VIII of the liver as well as further shrinkage of the aforementioned lesions (**Figure 1**). Due to the COVID-19 pandemic, the patient underwent the fourth to eighth pembrolizumab therapy at the local hospital. Subsequently, he underwent synchronous partial resection of the liver and partial right nephrectomy about 10 weeks ago (baseline + 36 weeks) with the pathology test result of the renal lesion indicated a clear-cell renal carcinoma while the immunohistochemistry test result showed CK (+), Vim (+), CD10 (+), CAIX (+), AMACR partial (+), PAX8 partial (+), Ki-67 (+) 3% (**Figure 2**). The posterior pancreas head metastatic lymph node involving pancreas and lower common bile duct was not resected because it was found complete colliquative necrosis and shrinked by 50% during the operation, thus a pancreatoduodenectomy was inevitable for complete resection, but the patient and his family refused to receive this procedure. Afterwards, the patient received the ninth pembrolizumab about 5 weeks ago (baseline + 41 weeks). The latest laboratory results showed a reduction in AFP and PIVKA-II levels to 0.95 μg/L and 21.32 mAU/mL. The latest CT scan showed that all the intrahepatic lesions had disappeared and the posterior pancreas head lymph node metastasis was completely necrosed and shrinked. As such, the patient was considered as complete response (CR) according to RECIST criteria after the comprehensive treatment.

## Discussion

HCC complicated with extrahepatic multiple primary cancer (MPC) is rarely reported, albeit high incidence rate of HCC. With the advancements in early cancer detection, the prevalence of MPC has increased gradually over the past decade. According to previous reports, the incidence rate of MPC ranged between 0.7 and 11.7% (6). The most common extrahepatic tumor site in patients with HCC complicated with MPC may vary from region to region. In Korea for instance, colorectal (30.3%), gastric (27.3%), and breast (12.1%) are the three most common extrahepatic sites involved in MPC patients (7). Whereas, in China, the most common extrahepatic sites refer to lung (15.0%), colorectal (12.5%) and thyroid gland (12.5%) (8). It is even rarer for MPC to occur in the liver and kidney at the same time. To date, only 9 such cases have been reported with none involving HCC complicated with extrahepatic lymph node metastasis (9–17). (**Table 1**)

**Table 1:** Reported Cases of Synchronous HCC and PRC.

Author	Age/Gender	Lesion	Treatment	Prognosis
Hou 2010 (15)	69/M	Single HCC (3*3*2.8cm), RCC (10*10*5cm)	Left radical nephrectomy and segmental resection of the liver	N/S
Shetty 2013 (9)	57/M	Single HCC (12×11cm), clear-cell RCC (4×3cm)	TACE,right hepatectomy and radical nephrectomy	No recurrence
Lee 2014 (16)	53/M	Single HCC (2.8cm), clear-cell RCC (1.9cm)	TACE and RFA	No recurrence after 1 month
Jekabsone 2014 (17)	58/M	Single HCC, clear-cell RCC (3×4×5cm)	Hepatic segmentectomy and radical nephrectomy	N/S
Gang 2015 (12)	72/M	Single grade I–II HCC and clear-cell RCC with renal vein invasion	Radiofrequency ablation and Sorafenib	Died, OS =40 weeks
Zhang 2015 (11)	72/M	Single HCC (10×9cm) with diaphragmatic and peritoneal invasion, clear-cell RCC (2×2cm)	TACE, right hemihepatectomy and partial wedge nephrectomy	No recurrence after 6 months
Sun 2016 (10)	42/M	Single HCC (15x8cm) with local invasion, clear-cell RCC (4×4cm)	Left hemihepatectomy and left nephrectomy	No recurrence after 4 months
Jiang 2020 (13)	47/M	Single HCC (3*4*5cm), clear-cell RCC (2*3*3cm) with right adrenal metastasis	Hepatic segmentectomy and right radical nephrectomy	No recurrence after 2 years
Ngo 2021 (14)	44/M	Single HCC (5×4cm), clear-cell RCC (1.5×1cm)	Left hemihepatectomy and radical nephrectomy	No recurrence after 10 months

M, male; N/S, not specified; HCC, hepatocellular carcinoma; RCC, renal cell carcinoma.

Although the pathogenesis of MPC remains unclear, the underlying mechanism was known to be attributed to host-, lifestyle-, environment-, gene- as well as treatment-related factors (18, 19). The risk factors of HCC include viral infection, alcohol intake, obesity, diabetes, and aflatoxin. Among others, Hepatitis C virus infection has been shown to have a significant association with an increased risk of renal cell carcinoma (20, 21).

After an in-depth discussion by the Molecular Tumor Board (MTB), our patient was diagnosed with HCC complicated with lymph node metastasis at posterior pancreas head, combined with PRC. Different from “HCC only” patients, management for second primary cancer should always be taken into careful consideration for HCC-associated MPC patients. As reported by 9 previous cases of HCC-associated PRC, all of them were solitary masses in both organs without lymph node metastasis. Seven out of the 9 cases received surgical treatment while the other 2 received interventional procedures and targeted therapy. Fortunately, all 9 patients achieved satisfactory outcomes after the treatments. However, our patient suffered from multifocal HCC with giant lymph node metastasis in the posterior pancreas head combined with PRC which has rendered the conventional radical resection implausible. Since there was no similar case reported in the past, the management for this patient is considered exploratory.

**Figure 3:**
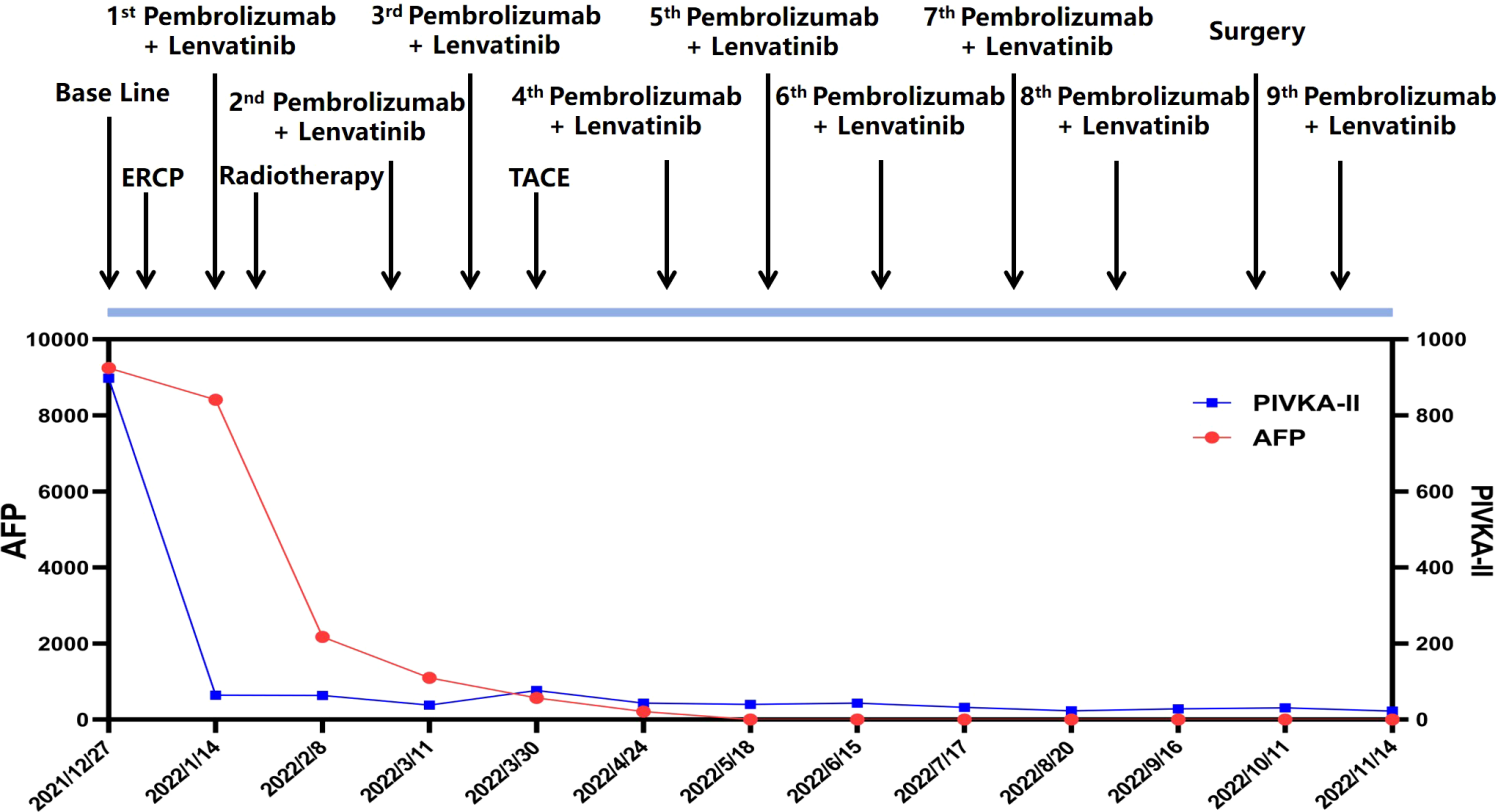
Timeline of the clinical course of the patient and the dynamic changes of tumor marker (AFP and PIVKA-II) along the treatment.

Due to the complexity of the case, MTB suggested to initiate combined antitumor therapy after the alleviation of biliary obstruction. Also, management of HCC was prioritized over PRC as recommended by urologists and oncologists due to higher malignancy portrayed by HCC. As per the recommendations of the National Comprehensive Cancer Network (NCCN) Guidelines on HCC, pembrolizumab and lenvatinib are preferred as the first-line therapy for advanced-stage liver cancer. And it is possible that the combination of pembrolizumab and lenvatinib can produce a synergistic antitumor effect (22, 23). Apart from that, NCCN Guidelines on PRC recommend the use of pembrolizumab plus lenvatinib as the first-line treatment for late-stage renal cancer (24). As a result of the combined treatment, significant shrinkage was appreciated in all lesions of the patient. (**Figure 3**)

The emergence of stereotactic body radiotherapy (SBRT) has also been playing an important role in preventing radiation-induced liver disease (RILD), the most dreadful complication of conventional radiotherapy (25). It does not only allow precise delivery of high dose radiation to HCC lesion, but also protects the normal liver tissue to ensure optimal liver function. What’s more, synergistic effect of SBRT can be observed in targeted therapy and immunotherapy by multiple mechanisms. Generally, repeated radiotherapy would in turn causes radioresistance in the tumor (26, 27). In this regard, targeted therapies are employed to modulate tumor cells and angiogenesis as well as promote ceramide-mediated cell apoptosis to create a radiosensitive microenvironment (28, 29). The combination of SBRT and PD-1 inhibitor can optimise antitumor immunity. SBRT promotes the release of tumor-associated antigens (TAAs) to overcome the immune evasion and resistance to PD-1 inhibitors (30, 31). Notably, SBRT can reduce the tumor burden by both direct killing and indirect immune effect that can further improve the therapeutic effect of PD-1 inhibitors (32).

For advanced HCC, TACE is often regarded as the first-line treatment (33–35), and TACE combined with lenvatinib has been shown to significantly improve the treatment efficacy and prolong overall survival (36). Therefore, TACE was also included in our management strategy to achieve a better survival benefit.

In conclusion, we report for the first case of multifocal HCC with giant lymph node metastasis in posterior pancreas head accompanied with PRC. His tumor biomarkers’ level were significantly reduced as well as the tumor sizes after comprehensive treatment including pembrolizumab plus lenvatinib, radiotherapy and TACE. Ultimately, the patient had successfully access to surgery and complete response of all the tumors were achieved. Pembrolizumab plus lenvatinib remains the maintenance therapy in the follow-up. Since there were no precedent cases and treatment guidelines for this type of MPC, we hope to provide a reference for diagnosis and treatment of similar patients in the future through our experience in this case.

## Data availability statement

The original contributions presented in the study are included in the article/Supplementary Material. Further inquiries can be directed to the corresponding author.

## Ethics statement

Written informed consent was obtained from the individual(s) for the publication of any potentially identifiable images or data included in this article.

## Author contributions

JC and ZZ performed the literature search and drafted the manuscript. XX designed and supervised the study. All authors contributed to the article and approved the submitted version.
